# The Role of Nitric Oxide and Reactive Oxygen Species in the Killing of *Leishmania braziliensis* by Monocytes from Patients with Cutaneous Leishmaniasis

**DOI:** 10.1371/journal.pone.0148084

**Published:** 2016-02-03

**Authors:** Pedro Paulo Carneiro, Jacilara Conceição, Michael Macedo, Viviane Magalhães, Edgar M. Carvalho, Olivia Bacellar

**Affiliations:** 1 Serviço de Imunologia, Hospital Universitário Professor Edgard Santos, Universidade Federal da Bahia, Salvador, Bahia, Brazil; 2 Instituto Nacional de Ciência e Tecnologia de Doenças Tropicais - INCT-DT (CNPq/MCT), Salvador, BA, Brazil; INRS - Institut Armand Frappier, CANADA

## Abstract

Human cutaneous leishmaniasis (CL) caused by *Leishmania braziliensis*, presents an exaggerated Th1 response that is associated with ulcer development. Macrophages are the primary cells infected by *Leishmania* parasites and both reactive oxygen species (ROS) and nitric oxide (NO) are important in the control of *Leishmania* by these cells. The mechanism involved in the killing of *L*. *braziliensis* is not well established. In this study, we evaluate the role of ROS and NO in the control of *L*. *braziliensis* infection by monocytes from CL patients. After *in vitro* infection with *L*. *braziliensis*, the oxidative burst by monocytes from CL patients was higher when compared to monocytes from healthy subjects (HS). Inhibition of the ROS pathway caused a significant decrease in the oxidative burst in *L*. *braziliensis* infected monocytes from both groups. In addition, we evaluated the intracellular expression of ROS and NO in *L*. *braziliensis*-infected monocytes. Monocytes from CL patients presented high expression of ROS after infection with *L*. *braziliensis*. The expression of NO was higher in monocytes from CL patients as compared to expression in monocytes from HS. A strong positive correlation between NO production and lesion size of CL patients was observed. The inhibition of ROS production in leishmania-infected monocytes from CL patients allowed the growth of viable promastigotes in culture supernatants. Thus, we demonstrate that while production of ROS is involved in *L*. *braziliensis* killing, NO alone is not sufficient to control infection and may contribute to the tissue damage observed in human CL.

## Introduction

Leishmaniasis is caused by protozoan parasites of the genus *Leishmania* transmitted by sandfly vectors. The promastigote form of the parasite invades host monocytes/macrophages and transforms into intracellular amastigotes. Host cells are able to control the infection if activated by IFN-γ. [1]. Human cutaneous leishmaniasis (CL) caused by *Leishmania braziliensis* is characterized by a strong cellular immune response and scarce numbers of parasites within lesions [2]. The presence of activating cytokines, such as IFN-γ and TNF, are important for control of parasite replication, but elimination of leishmania does not occur. The exaggerated Th1 immune response observed during *L*. *braziliensis* infection has been associated with severe inflammation and pathology [3–5].

During its life cycle, *Leishmania* encounters and readily adapts to various hostile conditions such as oxidative stress due to heme digestion in the blood meal, proteases in the sandfly midgut, complement-mediated lysis in the blood upon transmission, and reactive oxygen and nitrogen species (ROS and RNS) generated during phagocytosis by host macrophages [6–8]. Two important molecules that are critical in controlling *Leishmania* infection are superoxide anion (O_2_^-^) and nitric oxide (NO). During the initial phase of infection by *Leishmania*, superoxide is produced as part of the oxidative burst of macrophages in response to phagocytosis [6, 8]. The second oxidant produced by macrophages is nitric oxide, which, in contrast to superoxide, is generated after activation of macrophages by IFN-y and TNF [9], [10]. Nitric oxide is derived from the oxidation of the terminal guanidine nitrogen of L-arginine by an NADPH-dependent enzyme, NO synthase. In murine systems, IFN-γ has been shown to synergize with TNF, to activate inducible nitric oxide synthase (iNOS or NOS2) to produce nitric oxide (NO) resulting in eradication of intracellular parasites [11, 12].

In humans, several studies have shown iNOS is present in lesions of patients with cutaneous leishmaniasis caused by *Leishmania braziliensis* and *Leishmania tropica* [5, 13, 14], [15, 16]. Additionally, both iNOS mRNA and protein are expressed at high levels in bone marrow of patients with visceral leishmaniasis. iNOS protein was also found to be increased upon *in vitro* infection of human macrophages with *Leishmania chagasi* [9].

In monocytes from humans unexposed to *L*.*braziliensis*, we have shown that both promastigote and amastigote forms of *L*. *braziliensis* induce an oxidative burst but control of parasite replication is dependent on ROS [17]. We have previously shown that while macrophages from CL patients produce high amounts of pro-inflammatory cytokines, leishmania killing is impaired [17, 18]. Actually, patients with CL due to *L*. *braziliensis* produce high amounts of IFN-γ and TNF, but despite an environment adverse to leishmania proliferation, the infection progresses to disease. One possible explanation of this outcome includes impairment in the ability of monocytes in CL patients to develop an oxidative burst. Alternatively, it is possible that the oxidative burst produced by monocytes is insufficient to control leishmania. In this study, we showed that the oxidative burst is greater in CL monocytes than in healthy subjects (HS) monocytes. The increased oxidative burst is predominantly due to an increase in ROS rather than NO. However, while ROS production participates in leishmania killing, it is insufficient to prevent parasite multiplication. NO production, rather than control parasite growth, is associated with pathology.

## Material and Methods

### Patients

This study included 28 CL patients and 10 healthy subjects. All patients were examined at the Corte de Pedra clinic, in the state of Bahia, Brazil, a well-known region for *L*. *braziliensis* transmission. The criteria for diagnosis were a clinical picture characteristic of CL in conjunction with parasite isolation in culture or parasite identification in histopathologic analysis or an *L*.*braziliensis*-positive polymerase chain reaction result. Immunological studies were performed prior to therapy and 6 months to 1 year after cure (Table 1). All patients were treated with i.v. pentavalent antimonial (Sb^v^) (meglumine antimony; Sanofi-Aventis, Paris-France), 20 mg/kg body weight daily for 20 days. Criteria for cure were complete involution of lesions and/or total scarring of ulcers 90 days after initiation of treatment. Ten healthy subjects (HS) living in a place where there is no *L*. *braziliensis* transmission were enrolled in the study as controls. This research was conducted with approval of the Ethical Committee of the Professor Edgard Santos University Hospital, and informed consent was obtained from each participant.

**Table 1:** Clinical and epidemiological characteristics of cutaneous leishmaniasis patients (CL) and Healthy Subjects (HS).

	CL (n = 28)	HS (n = 10)
**Age (years)**	**30.5 (53–17)**	**30.3 (53–24)**
**Gender**	**19M; 6 F**	**2 M; 8 F**
**Lesion Size (mm)**	**14.8x13. 3**	**-**
**DTH reaction to leishmania antigen (mm)**	**14.3x14. 8**	**Negative**

### Parasite culture

An *L*. *braziliensis* (MHOM/BR/2003/LTCP11245) isolate obtained from a skin lesion of a CL patient was initially cultivated in biphasic medium (NNN). Following isolation, the parasites were cryopreserved in frozen nitrogen. The parasites selected for this study had not been previously passaged in liquid culture medium. After selection, the parasites were expanded in complete Schneider’s medium. The isolate was identified as *L*. *braziliensis* by multilocus enzyme electrophoresis [19]. All reagents and Schneider’s medium were endotoxin free as determined by Endotoxin Testing (LAL) (BioReliance, SIGMA-ALDRICH).

### Isolation of human peripheral blood cells and infection with *L*. *braziliensis*

Peripheral blood mononuclear cells (PBMC) were isolated from heparinized venous blood layered over a Ficoll-Hypaque gradient (GE Healthcare), washed, and resuspended in RPMI 1640 medium (supplemented with 5% of fetal calf serum, 100 U penicillin/mL, 100ug streptomycin/mL) (GIBCO BRL., Grand Island, NY USA). PBMC (1x10^6^ cells/tube) from CL patients and healthy subjects were infected with autologous serum opsonized *L*. *braziliensis* at a ratio of 5:1 cells at 37°C in 5% CO_2_. After 2 hours, extracellular parasites were removed after centrifugation. The cells were placed in complete RPMI 1640 medium and incubated for additional 24, 48 and 72 hours. In some experiments, monocytes were preincubated with either 10mM DPI (a specific inhibitor for flavoprotein, a constituent of the NADPH oxidase complex) or 1mM of NG-methyl-L-arginine acetate salt (L-NMMA), an inhibitor of nitric oxide synthase (iNOS) for 10 minutes and were infected with *L*. *braziliensis* promastigotes at a 5:1 ratio for 72 hours. After these periods of time, the number of infected cells and the number of intracellular parasites were determined by microscopic evaluation of 100 monocytes, after May-Grunwald-Giemsa staining from cytocentrifuge preparations.

### Evaluation of the oxidative burst

Isolated PBMC (1x10^6^) were stimulated with dihidrohodamine 123 at 10 ng/mL (Cayman Chemical Company) for 10 minutes at 37°C in a 5% of CO^2^. After that, cells were exposed to opsonized *L*.*braziliensis* promastigotes (ratio 5:1 cells) for 25 minutes at 37°C in a CO^2^ incubator. PMA (Phorbol 12-myristate 13-acetate) at 1 ug/mL was used as positive control for oxidative burst production. Monocytes were stained for surface markers with anti-CD14 (PerCP-Cy5.5 clone HCD14) and anti-CD16 (PE clone 3G8) and the fluorescence intensity of the cells assessed by flow cytometry and analyzed using FlowJo software. Monocytes were defined by nonspecific fluorescence with forward scatter (FSC) and side scatter (SSC) as parameters of cell size and granularity, respectively. The cells were then gated based on expression of CD14, CD16 and based on the oxidation of DHR 123 (S1 Fig).

The surface expression of TLR2 and TLR4 on monocytes from peripheral blood of CL patients and HS was analyzed both *ex vivo* and *in vitro* after infection with *L*. *braziliensis*. After incubation for 4 hours, analysis was performed by flow cytometry. The following antibodies were used: anti-CD14 conjugated with PerCP-Cy5.5 (clone 61D3); anti-TLR2 conjugated to PE (clone TL2.1) and anti-TLR4 PE-conjugated (clone HTA125) (IMGENEX, San Diego, CA, USA).

### Evaluation of oxidative burst after inhibition of enzymes NADPH oxidase and iNOS in monocytes after infection with *L*. *braziliensis*

To evaluate the effects of inhibition of ROS pathway or the NO pathway on the production of oxidative burst, cells were treated for 10 minutes with 10 mM DPI, a specific inhibitor for flavoprotein, a constituent of the NADPH oxidase complex. The inhibition of nitric oxide synthase (iNOS) was performed by treating monocyte cultures with 1mM of NG-methyl-L-arginine acetate salt (L-NMMA) for 10 minutes. Cells were then infected with *L*. *braziliensis* (5:1 cells) for 25 minutes at 37°C in 5% CO^2^. The oxidation of DHR-123 was assessed by flow cytometry.

### Intracellular production of NO and ROS by monocytes after infection with *L*.*braziliensis*

To detect specific intracellular NO production by monocytes, a fluorescent assay using the cell-permeable stain DAF-FM diacetate (4-amino-5-methylamino-2', 7'-difluorofluorescein diacetate) at 10 mM (Molecular Probe, Life Technologies) was used for 10 minutes before the infection with *L*. *braziliensis* promastigotes (5:1 cells). Intracellular ROS production by monocytes was measured by using an intracellular fluorescent probe, CM-H2DCFDA (carboxymethyl-H2-dichlorofluorescein diacetate) at 1μM (Molecular Probe, Life Technologies) for 10 minutes followed by infection for 25 minutes with *L*. *braziliensis* promastigotes (5:1 cells) at 37°C in a CO^2^ incubator. To evaluate the effects of the inhibition of ROS and NO pathway, DPI (10 mM, Sigma) and L-NMMA (1 mM) were added to cultures 10 minutes followed by infection for 25 minutes with *L*. *braziliensis* promastigotes (5:1 cells). Monocytes were stained for surface markers with anti-CD14 and anti-CD16 antibodies and the fluorescence intensity of the cells was assessed by flow cytometry and analyzed using Flowjo software.

### Viability of Parasites

After 72 hours of infection with *L*.*braziliensis*, monocytes were washed and the medium was replaced by 0.5 ml of Schneider’s medium (Sigma-Aldrich) supplemented with 10% fetal calf serum, to quantify the number of viable parasites. The tubes were cultured at 26°C for an additional 5 days. Viable number of *L*. *braziliensis* was estimated by proliferation of extracellular motile promastigotes in the Schneider's medium [20, 21].

### Evaluation of oxidative burst in patients after therapy and cure of disease

The production of oxidative burst in monocytes from CL patients was evaluated 1 year post treatment and complete cure of cutaneous leishmaniasis. The production of oxidative burst, NO and ROS after resolution of infection with *L*.*braziliensis* was determined by flow cytometry, as described above.

### Statistical Analyses

The data are presented as the mean ± SD of the mean. The significance of the results was calculated using nonparametric statistical tests: Mann-Whitney (two-sided *t* test), for comparisons between two groups or Kruskal-Wallis, followed by Dunn’s multiple comparison test, for comparisons between three groups. Correlation analysis was performed using Pearson´s correlation. All the analyses was conducted using Prism (GraphPad software) and a *p* < 0.05 was considered significant.

## Results

### Induction of the oxidative burst in monocytes from CL patients and HS after infection for *L*.*braziliensis*

**Fig 1:**
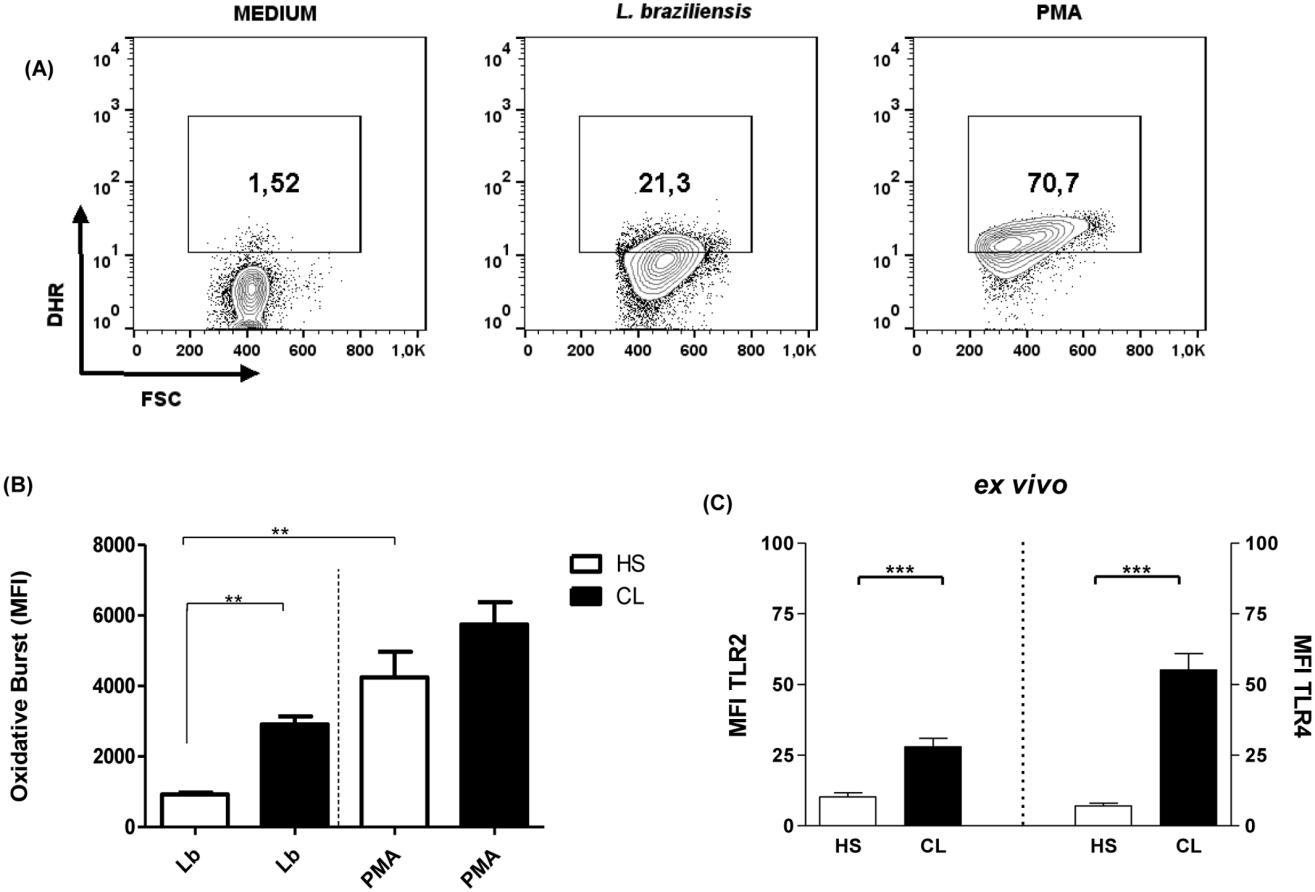
*L*.*braziliensis* induces high levels of oxidative burst in monocytes from CL patients. Monocytes from CL patients (n = 13) and HS individuals (n = 5) were treated with DHR (10ng/mL-10 min) and infected with *L*.*braziliensis* promastigotes for 25 minutes at a ratio of 5:1cells. PMA (1ug/ml) was used as positive control. Cells were stained with anti-CD14. Data were collected using flow cytometry and analyzed using FLOWJO software. (A) Representative gating strategy on CD14^+^ and DHR expression in monocytes from one CL patient (B) The data represent the median of mean intensity of fluorescence (MIF) of oxidative burst production by CL and HS monocytes. (C) The *ex vivo* expression of TLR2 and TLR4 was evaluated on CD14^+^ cells. Data were collected using flow cytometry and analyzed using FLOWJO software. Statistical analysis was performed using the Manny Whitney test and results were considered significant with a **p<0.01, ***p<0.001.

Monocytes (1x10^6^) from CL and healthy subjects (HS) were incubated with DHR-123 for 10 minutes before infection. The conversion of DHR-123 to rhodamine following oxidation was assayed by flow cytometry in monocytes cultures without stimulus, stimulated with PMA and after infection with *L*. *braziliensis* promastigotes (5:1 cells) for 25 minutes. The samples without stimulus and PMA treated were used to set the negative and positive regions for the fluorescence of the rhodamine, respectively. A fluorescence intensity (FL-1) higher than 10^3^ was interpreted as positive for intracellular oxidative burst (Fig 1a).

We first assessed the oxidative burst in monocytes from CL patients after infection with *L*. *braziliensis* compared with the production by monocytes from HS. After infection with *L*. *braziliensis*, the oxidative burst by monocytes from CL patients represented by the mean fluorescence intensity (MFI), 2821 ± 1040 was higher than the MFI in monocytes from HS 1091 ± 268 (p <0.01) as shown in Fig 1b. There was no difference in the oxidative burst in unstimulated cells from CL and HS (243 ± 102 *versus* 172 ± 74) and in cells stimulated with PMA (4240 ± 1635 *versus* 5741 ± 2294).

As Toll-like Receptor (TLR) expression has been associated with oxidative burst [22, 23] and can be induced upon leishmania infection, we evaluated the *ex vivo* expression of TLR2 and TLR4 (Fig 1c). Both TLR2 and TLR4 were more highly expressed *ex vivo* in monocytes from CL (28 ± 9 and 55 ± 18) than HS (10 ± 5 and 7 ± 3), respectively (p<0.001). After *ex vivo* infection with *L*.*braziliensis*, the expression of TLR2 and TLR4 increased significantly in monocytes isolated from CL patients (32 ± 26 *versus* 90 ± 64, and 14 ± 7 *versus* 55 ± 32, respectively), p<0.001.

### Expression of oxidative burst in *L*. *braziliensis* infected monocytes after inhibition of NADPH oxidase and iNOS enzymes

To determine the importance of ROS and NO in the generation of oxidative burst in monocytes during CL, infection assays were conducted in the presence of inhibitors of nitric oxide synthase (L-NMMA) and of NADPH oxidase (DPI).

**Fig 2:**
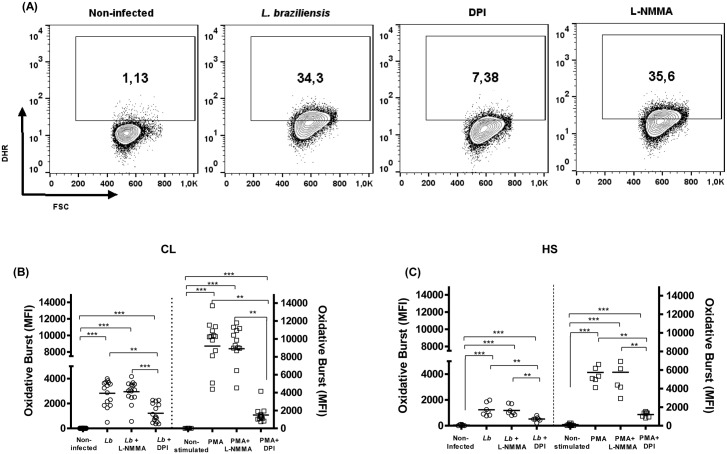
Inhibition of NADPH oxidase decreases the oxidative burst. Monocytes from CL patients (n = 15) and HS individuals (n = 7) were preincubated with either DPI (10mM), an inhibitor of the NADPH oxidase, or L-NMMA (1mM), an iNOS inhibitor, for 10 minutes. The monocytes were pre-incubated with DHR (10 minutes) and infected with *L*.*braziliensis* promastigotes (5:1cells) or stimulated with PMA (1 ug/mL) for 25 minutes. Cells were stained with anti-CD14. Data were collected using flow cytometry and analyzed using FLOWJO software. (A) Representative contour plots. (B) The data represent the median of mean intensity of fluorescence (MIF) of DHR expression by monocytes from CL patients and HS individuals (C). Statistical analysis was performing using ANOVA with Bonferoni´s pos-test and Manny Whitney test. The results were considered significant with a p< 0.05 (** p<0.01; ***p<0.001).

Preincubation with DPI (10 mM for 10 minutes) caused a significant decrease in the oxidative burst in *L*. *braziliensis* infected monocytes (5:1 cells for 25 minutes) from CL patients (2821 ± 1040 *versus* 1225 ± 724), (P<0.001). L-NMMA pretreatment (1 mM for 10 minutes) did not alter the oxidative burst in *L*. *braziliensis* infected monocytes from CL patients (2948 ± 879 *versus* 2821 ± 1040) (Fig 2a). In cells from HS, the addition of DPI also decreased the oxidative burst (1239 ± 494 *versus* 514 ± 175), (p <0.05) while L-NMMA had no effect. Monocytes stimulated with PMA (1ug/mL for 25 minutes) in the presence of DPI or L-NMMA showed similar results to those observed after infection with *L*. *braziliensis* promastigotes (Fig 2a and 2b). The oxidative burst (DHR123 MFI) in uninfected or unstimulated cells was 34 ± 32 and 46 ± 45 respectively. The addition of DPI and LNMMA to uninfected or unstimulated cells did not change these data.

These results suggest that oxidation of rhodamine in monocytes from patients with CL and HS after infection by *L*. *braziliensis* or stimulation with PMA reflects an increased production of reactive oxygen species during the oxidative response.

### Intracellular production of ROS and NO by monocytes from CL patients and HS after infection of *L*. *braziliensis*

**Fig 3:**
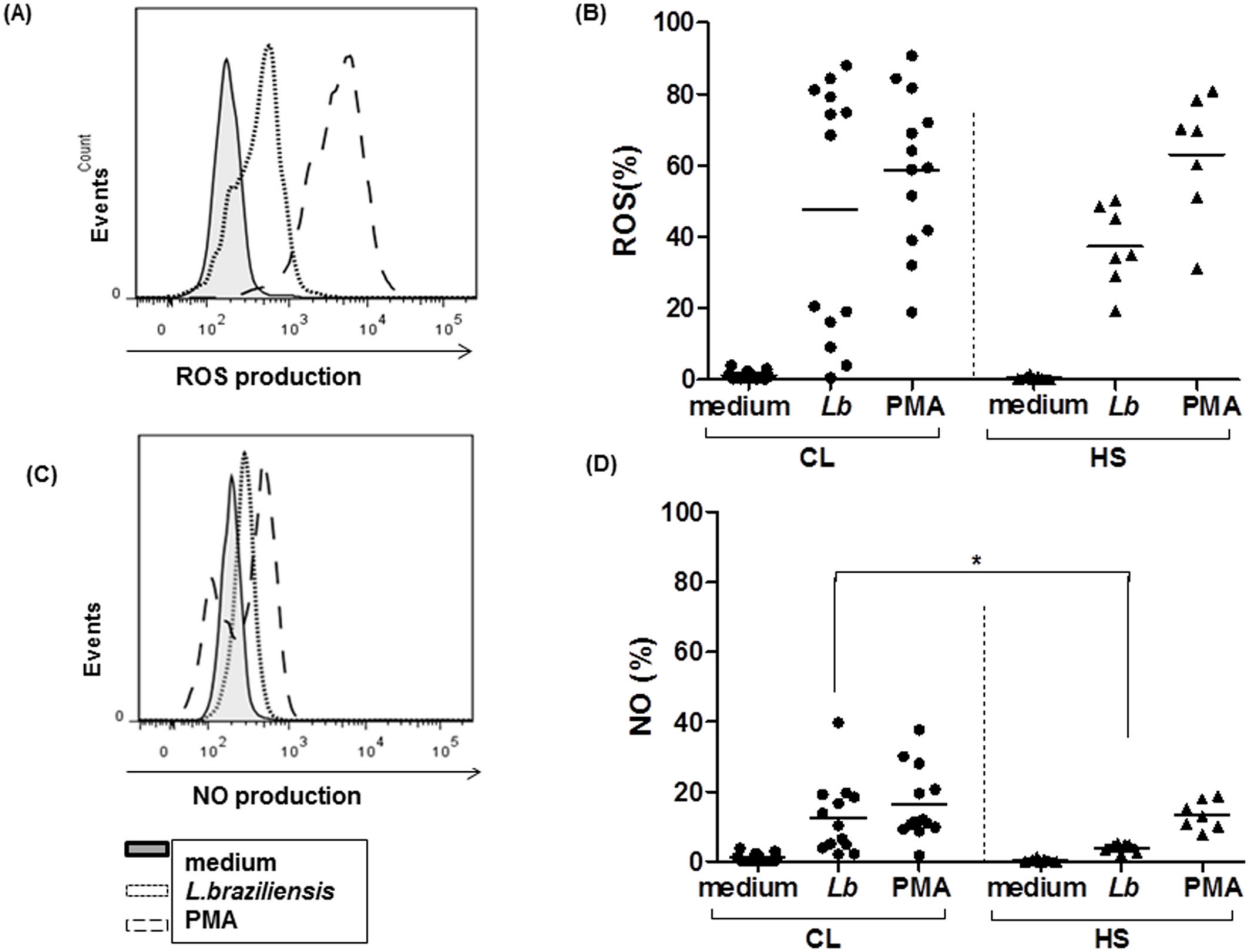
Monocytes from CL patients produced high levels of reactive oxygen species after infection with *L*.*braziliensis*. Monocytes from CL patients (n = 13) and HS individuals (n = 7) were stained with DAF-FM diacetate (NO probe, 10mM) and CMH-2DCFDA (ROS probe, 1 μM) for 10 minutes, infected with *L*.*braziliensis* promastigotes for 25 minutes at a ratio of 5:1cells, and stained with anti-CD14. PMA was used as positive control. Data were collected using flow cytometry and analyzed using FLOWJO software (A). Representative histograms of ROS production, (B) Frequency of *L*.*braziliensis*-infected monocytes expressing ROS, (C) Representative histograms of NO production, (D) Frequency of *L*.*braziliensis*-infected monocytes expressing ROS. Statistical analysis was performing using ANOVA with Bonferoni´s pos-test and Manny Whitney test. The results were considered significant with a p< 0.05 (*p<0.05).

Our data provides evidence that ROS is more induced than NO during the oxidative response by monocytes after infection with *L*. *braziliensis*. To further elucidate these data, we measured intracellular production of ROS and NO after infection with leishmania, using CM-H2DCFDA (1 μM for 10 minutes), an indicator of intracellular ROS production [24] and DAF-FM diacetate (10 mM for 10 minutes), an indicator of NO production [25]. The frequency of monocytes from CL patients expressing ROS after infection with *L*. *braziliensis* promastigotes (5:1 cells for 25 minutes) was higher (47 ± 35%) than those observed in cells from HS (37±11%), although without statistical significance. However, the production of NO by cells from CL patients after infection was higher (p<0.05) than in HS (12 ± 10% *versus* 3.9 ± 1.2%), (Fig 3a and 3b).

### The ability of monocytes from CL patients and HS in control *L*.*braziliensis* infection

**Fig 4:**
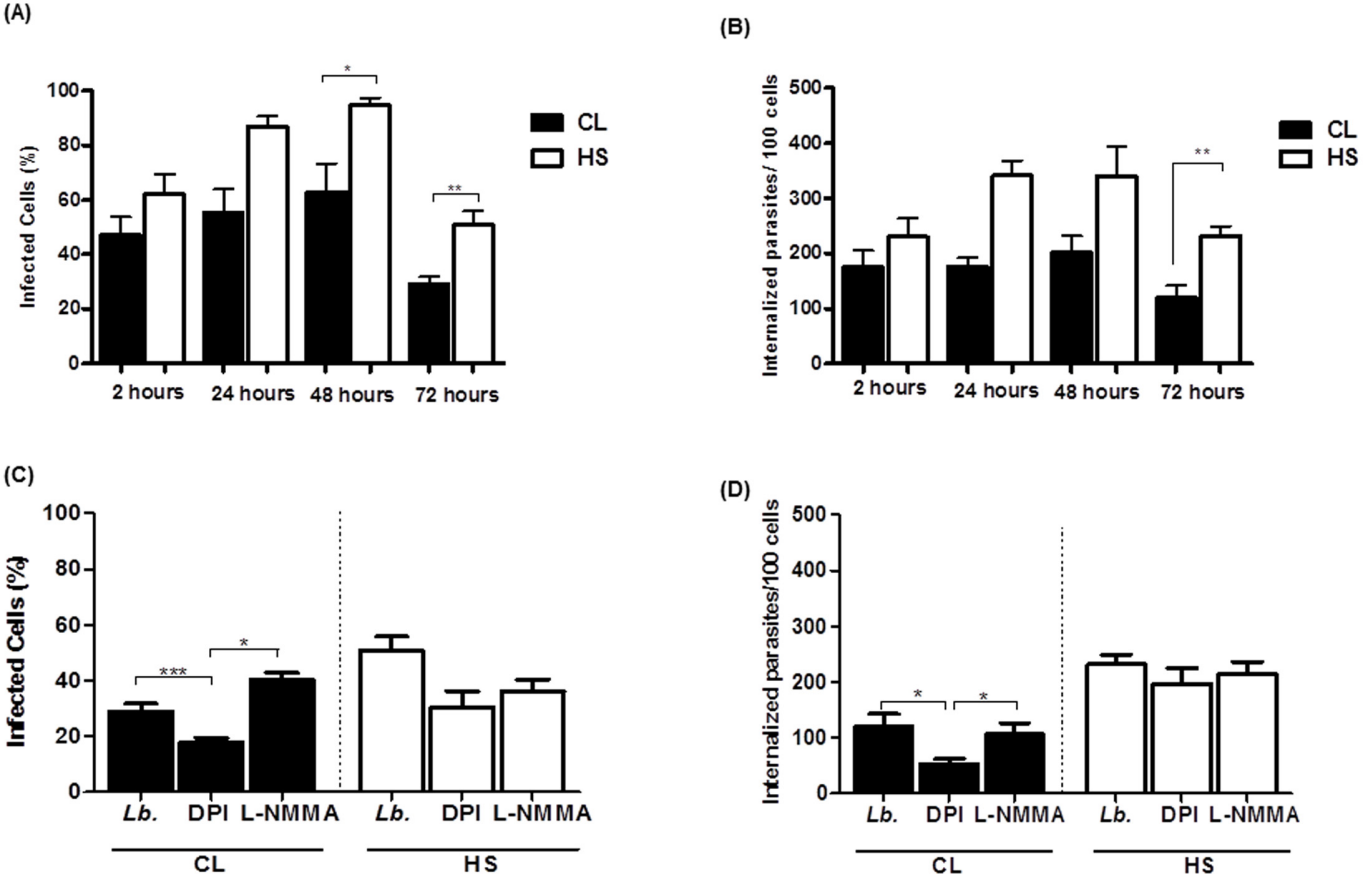
Phagocytosis and the killing of *L*. *braziliensis* by monocytes from CL patients. Monocytes from CL patients (n = 9) and HS individuals (n = 6) were infected with *L*. *braziliensis* promastigotes at a 5:1 ratio for 2, 24, 48 and 72 hours. The number of infected cells (A) and the number of intracellular parasites (B) were determined by microscopic evaluation after May-Grunwald-Giemsa staining from cytocentrifuge preparations. Monocytes were preincubated with either DPI (10mM) or L-NMMA (1mM), for 10 minutes and were infected with *L*. *braziliensis* promastigotes at a 5:1 ratio for 72 hours. (C) The number of infected cells. (D) The number of intracellular parasites. Statistical analysis was performed using the Kruskal-Wallis test (* p < 0.05, ** p < 0.01).

We infected monocytes from CL patients and HS with *L*. *braziliensis* for different periods of time. After cytocentrifugation, the number of infected cells and the parasite load were evaluated using light microscopy. The percentage of infected cells and the number of intracellular amastigotes/100 monocytes were similar in the groups 2h after infection. The percentage of cells infected with *L*.*braziliensis* and the number of parasites/100 monocytes was lower in monocytes from CL patients as compared to monocytes from HS. The percentage of infected cells was also lower at 24, 48 and 72 hours in monocytes from CL patients than in HS monocytes (55 ± 22 *versus* 87 ± 8; 62 ± 22 *versus* 94 ± 6; and 29 ± 7 *versus* 50 ± 11) (p<0.05), as shown in Fig 4a. After 48 and 72 hours, the parasite load was also lower in monocytes from CL patients as compared to HS (176 ± 40 *versus* 341 ± 119; and 119 ± 61 *versus* 232±38), p<0.05, p<0.01 (Fig 4b).

At the time of 2, 24 and 48 hours there was no difference in the number of infected cells or number of internalized parasites in the presence of DPI or LNMMA in monocytes from untreated CL or HS cells (S2 Fig). However, at 72 hours, in monocytes from CL patients, the number of infected cells (Fig 4c) and the number of internalized parasites (Fig 4d) was lower in the presence of DPI (29 ± 7 *versus* 17 ± 4; and 119 ± 61 *versus* 53 ± 23), p<0.001 and p<0.05. A possible explanation for this observation is that the inhibition of ROS production allows the parasite survival. Together these data suggest that the production of ROS by monocytes from CL patients may increase the ability of these cells to kill leishmania.

### Evaluation of the viability of *L*. *braziliensis* promastigotes after inhibition of NO and ROS pathways

**Fig 5:**
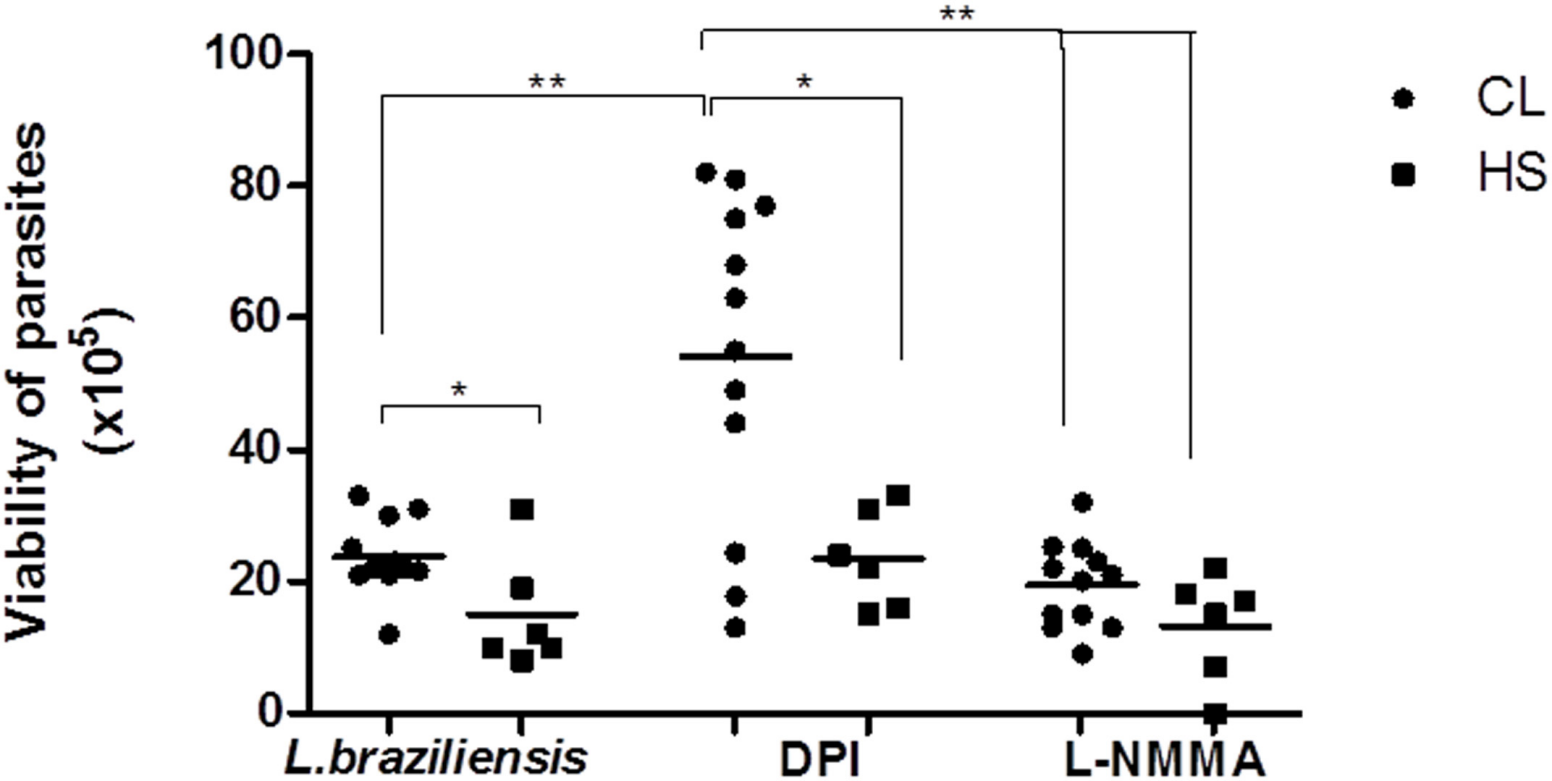
The role of NO and NO in the control of *L*.*braziliensis* infection by monocytes from CL patients. Monocytes from CL patients (n = 9) and HS individuals (n = 6) were treated with inhibitor of the NADPH oxidase (DPI-10mM) or with an iNOS inhibitor (L-NMMA-1mM) for 10 minutes and infected with *L*.*braziliensis* at a 5:1. After 72 hours, the medium of monocytes culture was replaced by Schneider’s medium and after 5 days the number of viable promastigotes was estimated. Statistical analysis was performed using the Manny Whitney test. (*** p < 0.001).

To assess whether the decrease in the parasite load was related to leishmania killing, we evaluated the viability of promastigotes in cultures of infected monocytes from CL patients in the presence or absence of oxidant inhibitors. The number of viable promastigotes, estimated by proliferation of extracellular motile parasites in Schneider´s medium, was higher in cultures of monocytes from CL patients (23 ± 5.6) as compared to monocyte from HS (15 ± 8.7), p<0.05 (Fig 5). In the presence of ROS inhibitor, the number of viable *L*. *braziliensis* was higher (66 ± 14) as compared with infected monocytes alone (21 ± 8) or in the presence of NO inhibitor (18 ± 7), p<0.001 (Fig 5). However, in cultures of infected monocytes from HS, we did not observe any difference in number of viable parasites in the presence of inhibitors. The reduced number of internalized parasites at 72 hours shown in Fig 4 is compatible with the higher number of viable promastigotes observed in supernatants of culture. What may be happening is that intense intracellular proliferation of parasites causes disruption of the cell, allowing the release of live parasites into the supernatant.

These results indicate that in CL patients, production of ROS participates in parasite killing, but despite its production a high percentage of monocytes remain infected and a large amount of amastigotes are found in such cells.

### Correlation between oxidant production by *L*. *braziliensis* infected monocytes and lesion size in CL patients

**Fig 6:**
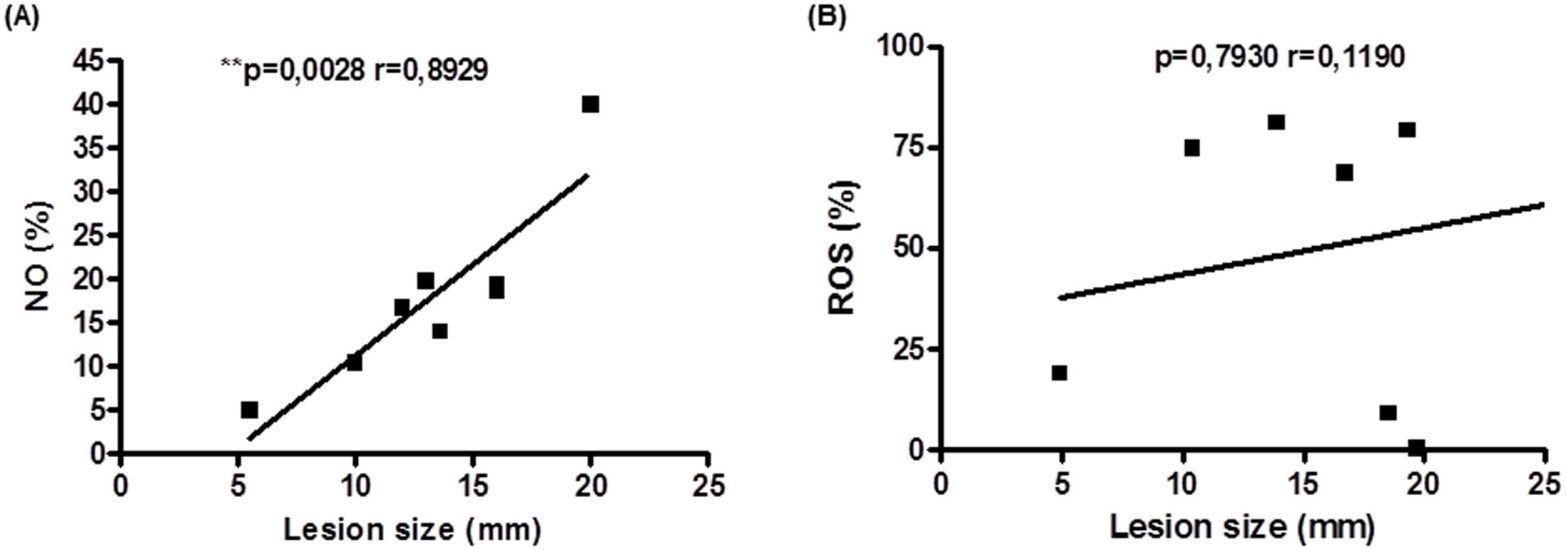
Correlation between NO and ROS production by monocytes and lesion size of CL patients. Monocytes from CL patients (n = 8) were treated with DAF-FM diacetate (NO probe, 10mM) or CMH-2DCFDA (ROS probe, 1 μM) for 10 minutes and infected with *L*.*braziliensis* promastigotes for 25 minutes at a ratio of 5:1cells as described in materials and methods. Production of NO and ROS was evaluated by flow cytometry. (A) Correlation between NO production (%) and lesion size (mm). (B) Correlation between ROS production (%) and lesion size (mm). Statistical analysis was performed using the Pearson correlation.

The production of NO has been implicated in the pathogenesis of several inflammatory diseases, such as tuberculoid leprosy and psoriasis [26–28]. In cutaneous leishmaniasis caused by *Leishmania mexicana*, iNOS expression was correlated with increased number of skin lesions [29]. We extend our observations showing that in *L*. *braziliensis* infection, there is a positive correlation between NO production by monocytes and lesion size in CL patients (Fig 6a). On the other hand, no correlation between production of ROS and lesion size was observed (Fig 6b).

### The production of NO and ROS by monocytes of CL patients after therapy of American Tegumentary Leishmaniasis

**Fig 7:**
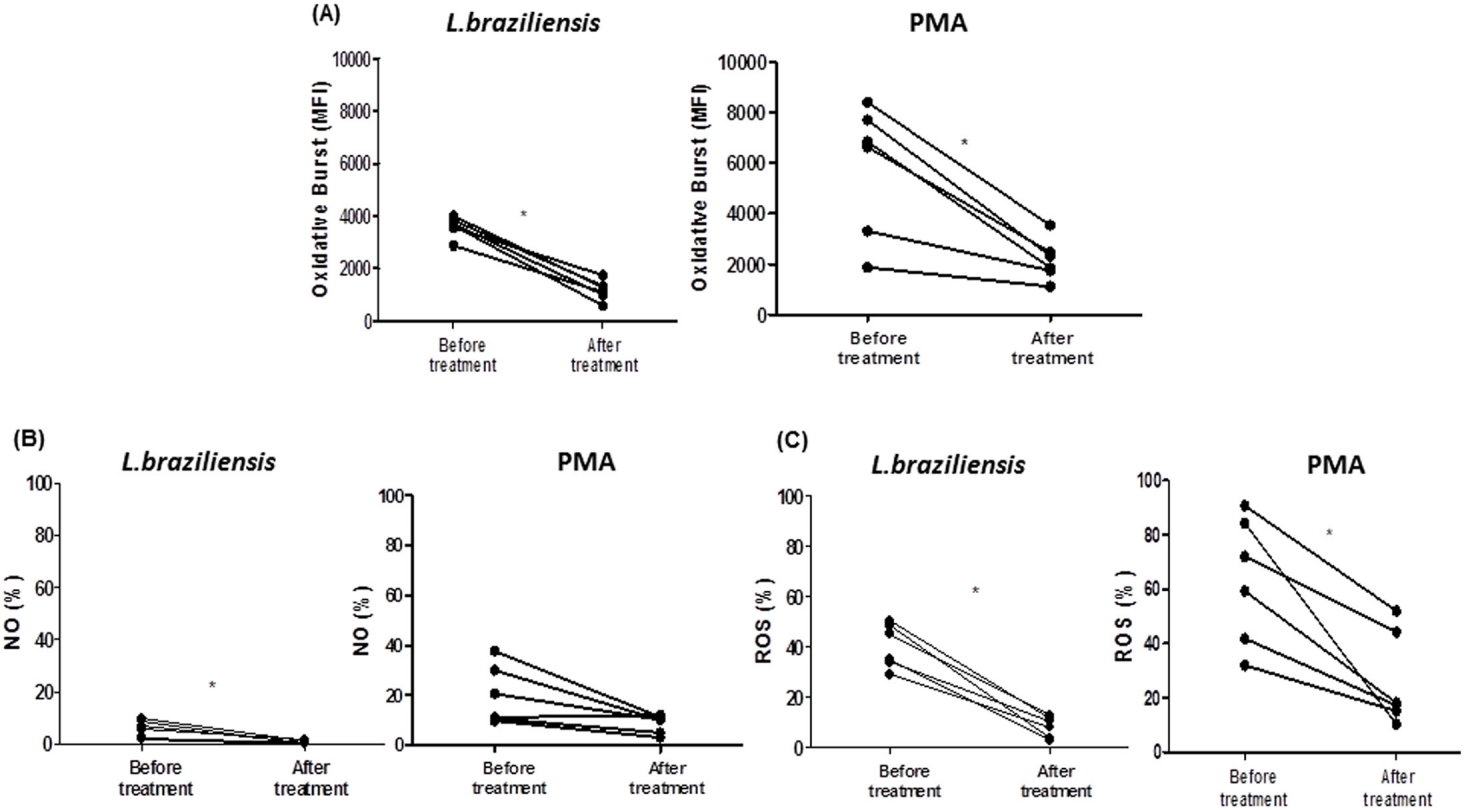
Oxidative burst production before and after therapy and cure of CL patients. Production of burst oxidative, NO and ROS by monocytes from CL patients (n = 6) after infection with *L*.*braziliensis* promastigotes or upon PMA stimulus, were determined before and after therapy (i.v. pentavalent antimonial, 20mg/kg body weight daily for 20 days) and cure of cutaneous leishmaniasis. The data represent the median of mean intensity of fluorescence (MIF) of oxidative burst production (A), frequency of NO production (B) and frequency of ROS production (C). Statistical analysis was performed using Wilcoxon test and results were considered significant (p<0.05).

All patients were treated with i.v. pentavalent antimonial, 20 mg/kg body weight, daily for 20 days. Criteria for cure were complete involution of lesions and/or total scarring of ulcers 90 days after initiation of treatment. After treatment and cure of patients with cutaneous leishmaniasis, the production of NO and ROS was evaluated in monocytes after *L*.*braziliensis* infection (Fig 7). There was a significant decrease in production of oxidative burst by these cells after therapy 3603±160 *versus* 1179±157, p<0.05 (Fig 7a). A decrease in NO and ROS levels was also observed, 5.8±1.3 *versus* 0.705 ± 0.259 and 40.3±3.5 *versus* 8.4 ±1.6, respectively, p <0.05 (Fig 7b and 7c). The cure is associated with the decreased or eradication of the parasite and also associated with a decreased inflammatory response. Alternatively, it has been reported that antimony treatment can induce ROS and NO generation to kill leishmania [30]. Thus, we believe that the successful therapy contributes to decreased oxidative burst. This observation indicates that the high inflammatory response observed in CL regulates ROS and NO production.

## Discussion

Monocytes play a critical role in leishmania infection, not only are they a primary leishmania host cell but also the main cell with the ability to eradicate parasites. Human CL is characterized by an exaggerated Th1 immune response, but despite the production of high levels of IFN-γ and TNF, the parasites survive. Compared with cells from subclinical *L*. *braziliensis* infected subjects (SC), CL monocytes/macrophages are more permissive to parasite growth and eliminate fewer parasites than cells from SC subjects [18, 31]. We evaluated whether or not *L*. *braziliensis* infection was able to induce the oxidative burst in monocytes from CL patients, and the production and role of ROS and NO in parasite killing. Our data indicate that *L*. *braziliensis* induce a greater oxidative burst in CL than in HS monocytes. This observation could be due to the inflammatory environment of patient’s cells and the greater expression of TLR2 and TLR4 on monocytes from CL patients. This increase in oxidative burst by monocytes is mainly related to ROS, a molecule involved in parasite eradication. Moreover, NO was also enhanced after *L*. *braziliensis* infection, but while NO production was not associated with parasite killing, there was a direct correlation between NO production and the size of the CL ulcer. In post kala-azar dermal leishmaniasis (PKDL), caused by *Leishmania donovani*, production of NO in cultured cells stimulated with leishmania antigen was elevated compared with post treatment samples, suggesting that this molecule may be associated with the pathogenesis of PKDL [32].

The oxidative burst is the main mechanism used by macrophages for leishmania eradication and O_2_^-^ and NO are the main molecules utilized by the oxidative burst [9, 33]. As expected, after infection with *L*. *braziliensis*, unstimulated monocytes and PMA stimulated cells showed a similar increase in oxidative burst in HS and CL monocytes. These results suggest that the parasite may induce a stronger oxidative burst in monocytes from CL patients.

Factors involved in ROS production include phagocytosis and TLR expression [22, 34]. Therefore, increased oxidative burst in CL monocytes could be due to increased leishmania uptake by CL monocytes or increased expression of TLRs. Penetration and uptake of *L*. *braziliensis* was similar between CL and HS monocytes. However, uninfected monocytes from CL had increased TLR-2 and TLR-4 expression compared with HS monocytes. Also, infection with *L*. *braziliensis* significantly increased the TLR-2 and TLR4 expression on monocytes in CL patients. Several studies have shown a role for TLRs in the generation of oxidative burst during leishmania infection [23, 35]. For instance, Srivastava et al. demonstrated that higher expression of TLR2 was associated with a higher oxidative response and increased iNOS expression in macrophages infected with *L*. *major* [36].

The role of NO in the defense mechanisms of human monocytes/macrophages is controversial and may be specific to the leishmania species. We have previously shown that the percentage of infected monocytes, as well the number of amastigotes of *L*. *braziliensis*, were similar in monocytes stimulated with IFN-γ and treated with or without L-NMMA, a nitric oxide synthesis inhibitor. However, treatment of IFN-γ stimulated cells with N-acetyl cysteine (NAC), an ROS scavenger, increased parasite multiplication [17]. In this study, we extend these observations showing that *L*. *braziliensis*-infected monocytes from CL patients produce more ROS than monocytes from HS and treatment with L-NMMA did not increase parasite growth. Further, both forms of *L*. *braziliensis* (promastigotes and amastigotes) induced the oxidative burst in monocytes from CL patients and HS (data not shown), as previously demonstrated [20]. Concurrently, we found a strong correlation between production of NO and lesion size of CL patients. These results suggest that, while NO production alone does not participate in the control of infection, it may contribute to the tissue damage observed in human CL.

To evaluate the ability of monocytes from CL patients to kill *L*. *braziliensis* and whether or not killing could be associated with the oxidative burst, we determined the frequency of infected monocytes at different times, the numbers of amastigotes inside the cells and parasite viability in supernatants of *L*. *braziliensis* infected monocytes. Despite CL monocytes displaying greater oxygen burst than HS cells, there was not a dramatic decrease in the percentage of infected cells, although a few amastigotes were detected through 24 to 72 hours inside of CL monocytes. Moreover, the parasite viability was similar in the supernatants of CL and HS monocytes infected with *L*. *braziliensis*, but the parasite viability was increased in supernatants of CL monocytes treated with DIP, a ROS inhibitor, indicating the role of this molecule in parasite killing. However, the addition of DIP did not affect the parasite viability in supernatants of HS monocytes indicating that ROS did not affect parasite survival in some infected monocytes. The reason why, despite greater respiratory burst, the parasites survive is not clear. One possibility could be the use of evasion mechanisms as production of superoxide dismutase [37, 38]. Alternatively, it is possible that some monocytes produce lower amounts of ROS. The monocytes are a heterogeneous population and based on the expression of CD14 and CD16, it can be classified as classical, intermediate or inflammatory and non-classical monocytes [39]. While in *L*. *braziliensis*, infection there is an increase in the frequency of intermediate monocytes and production of pro-inflammatory cytokines [40], *L*. *braziliensis* killing is mediated by classical monocytes [17]. We have not determined the ROS production in monocyte subsets but it cannot be ruled out that some monocyte subsets may have different expressions of oxygen burst than others. The persistence of parasites on monocytes and macrophages stimulates and contributes to maintenance of the inflammatory reaction that leads to ulcer development.

Due to *L*. *braziliensis*, CL has an inflammatory environment with high production of Th1 cytokines, IL-17 and pro-inflammatory chemokines [18, 41–43]. This inflammatory environment contributes to the increase in respiratory burst in *L*. *braziliensis* infected cells, as successful therapy was followed by a significant reduction in the oxidative burst. The participation of the inflammatory response in the pathogenesis of CL ulcers has been well documented. Progression from infection to disease occurs despite IFN-γ and TNF production and there is a direct correlation between the expression of TLR9 and lesion size and a positive correlation between the frequency of cell expressing IFN-γ cell, TNF as well as T cell activation markers, and the lesion size [4, 44, 45]. Moreover, molecules that down modulate the immune response as GM-CSF and pentoxyfilline are more effective than antimony alone in curing CL ulcers, reducing the healing time and in curing patients that are refractory to antimony therapy [46, 47]. In conclusion, monocytes from CL display greater expression of the oxidative burst, and despite the role of ROS in parasite control by these cells, it is not sufficient to kill *L*.*braziliensis* from infected cells. Alternatively, while production of NO does not participate in leishmania eradication, it may contribute to the tissue damage observed in human CL.

## Supporting Information

S1 Fig Representative plots used for the analysis of monocyte expressing CD14 and oxidative burst production. Peripheral blood mononuclear cells (PBMC) were obtained and stimulated with dihidrohodamine 123 (DHR) for 10 minutes. Monocytes were infected with *L*. *braziliensis* at a 5:1 ration for 20 minutes and stained for CD14 as indicated on materials and methods.
(TIF)

S2 Fig Phagocytosis and killing of *L*. *braziliensis* by monocytes from CL patients after ROS and NO inhibition. Monocytes from CL patients (n = 9) and HS individuals (n = 6) were infected with *L*. *braziliensis* promastigotes at a 5:1 ratio for 2, 24, 48 and 72 hours. Monocytes were preincubated with either DPI (10mM) or L-NMMA (1mM), for 10 minutes and were infected with *L*. *braziliensis* promastigotes at a 5:1 ratio for 72 hours. The number of infected cells (A and C) and the number of intracellular parasites (B and D) were determined by optical microscopy. Statistical analysis was performed using the Kruskal-Wallis test (* p < 0.05, ** p < 0.01).
(TIF)

## Abbreviations

CL: Cutaneous Leishmaniasis
HS: Healthy Subjects
ROS: Reactive Oxygen Species
NO: Nitric Oxide
TLR: Toll Like Receptor
PMA: Phorbol 12-myristate 13-acetate
DHR: dihidrohodamine
FSC: Forward scatter
SSC: Side scatter
DPI: Diphenyleneiodonium
L-NMMA: NG-methyl-L-arginine acetate salt
CM-H2DCFDA: Carboxymethyl-H2-dichlorofluorescein diacetate
DAF-FM diacetate: 4-amino-5-methylamino-2 ', 7'-difluorofluorescein diacetate
