# Supplementary figures and images for: The Role of Nitric Oxide and Reactive Oxygen Species in the Killing of Leishmania braziliensis by Monocytes from Patients with Cutaneous Leishmaniasis
Source: PLoS One. 2016 Feb 3;11(2):e0148084. doi: 10.1371/journal.pone.0148084 (PMC4739692; doi:10.1371/journal.pone.0148084)

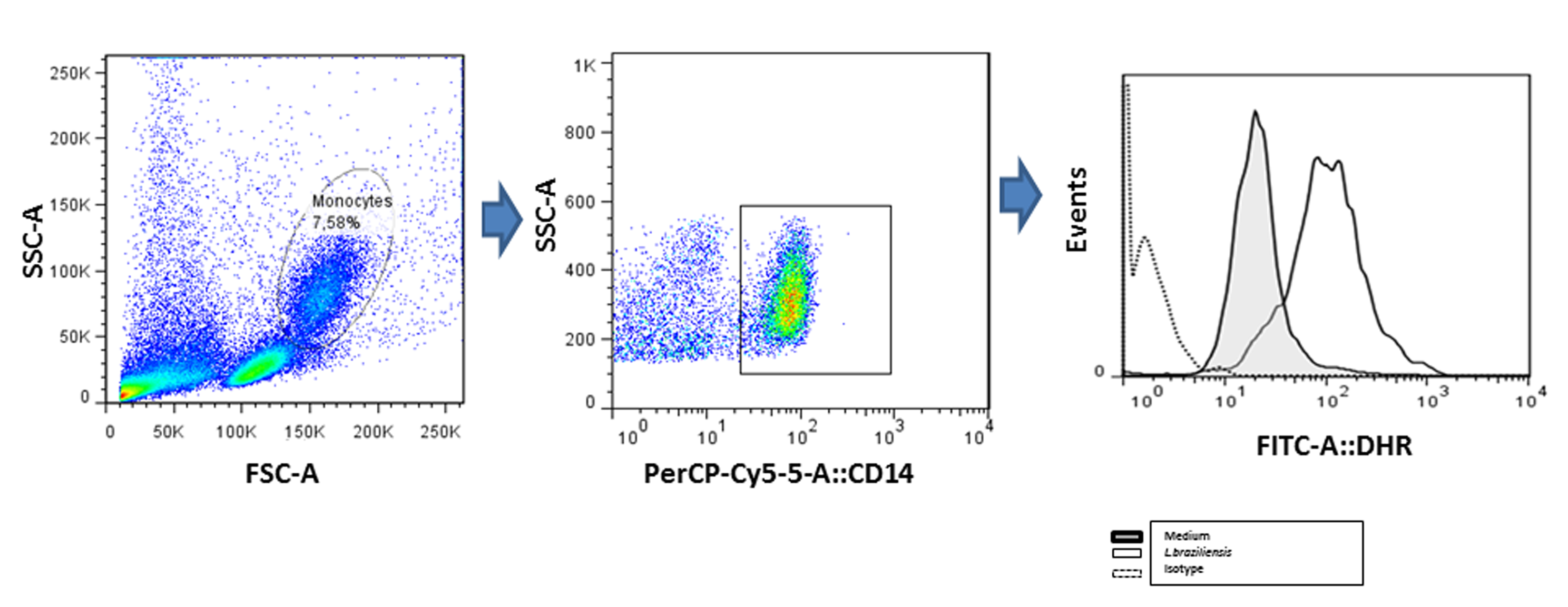

Supplement: S1 Fig — Peripheral blood mononuclear cells (PBMC) were obtained and stimulated with dihidrohodamine 123 (DHR) for 10 minutes. Monocytes were infected with L. braziliensis at a 5:1 ration for 20 minutes and stained for CD14 as indicated on materials and methods. (TIF) [file pone.0148084.s001.tif]

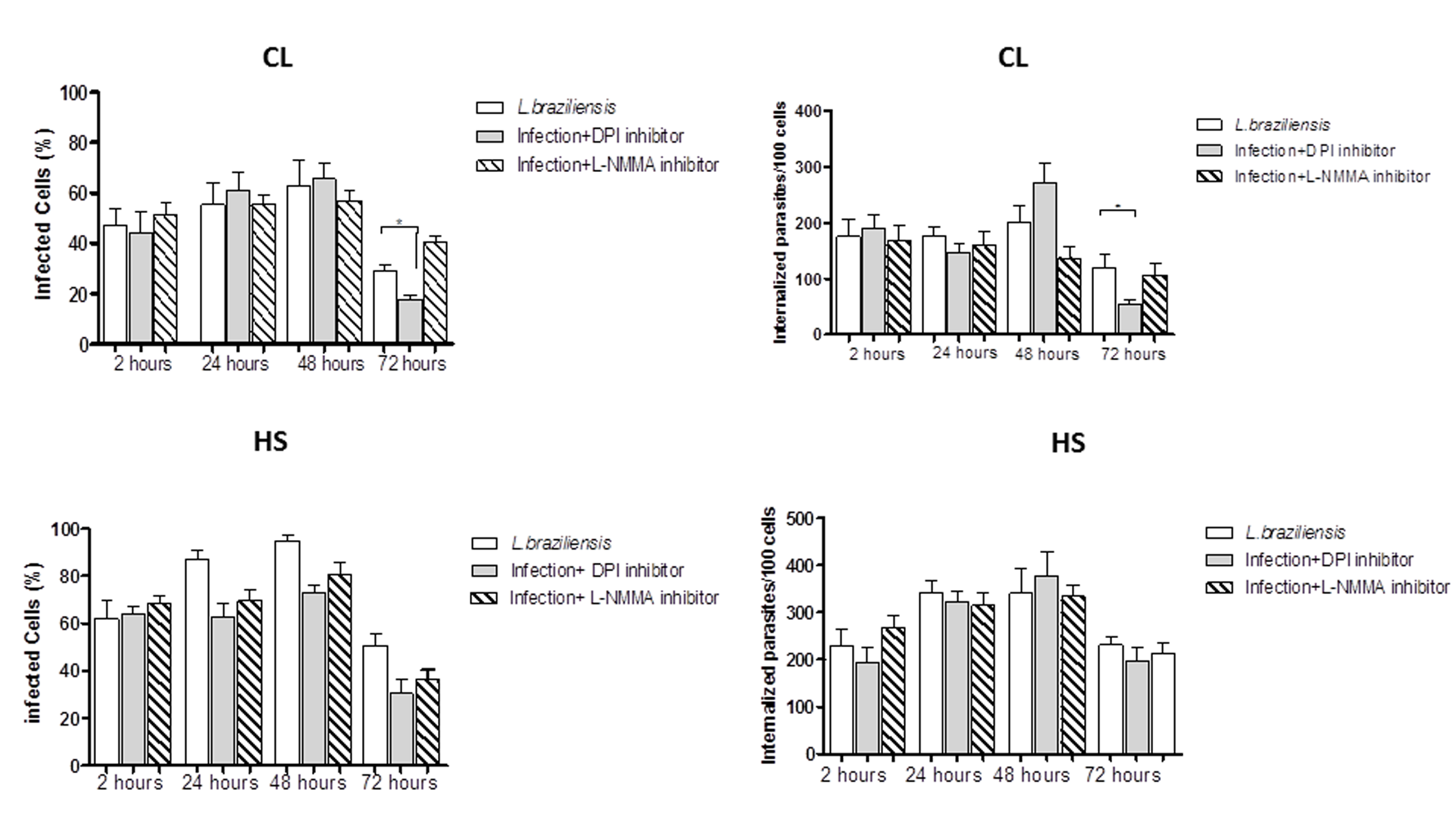

Supplement: S2 Fig — Monocytes from CL patients (n = 9) and HS individuals (n = 6) were infected with L. braziliensis promastigotes at a 5:1 ratio for 2, 24, 48 and 72 hours. Monocytes were preincubated with either DPI (10mM) or L-NMMA (1mM), for 10 minutes and were infected with L. braziliensis promastigotes at a 5:1 ratio for 72 hours. The number of infected cells (A and C) and the number of intracellular parasites (B and D) were determined by optical microscopy. Statistical analysis was performed using the Kruskal-Wallis test (* p < 0.05, ** p < 0.01). (TIF) [file pone.0148084.s002.tif]
